# Supplementary material for: Dynamic Changes in the Global Transcriptome of Postnatal Skeletal Muscle in Different Sheep
Source: Genes (Basel). 2023 Jun 20;14(6):1298. doi: 10.3390/genes14061298 (PMC10297920; doi:10.3390/genes14061298)
Supplement: Supplementary file 1 [file genes-14-01298-s001.zip › FigureS2.pdf]

Figure. S2

A

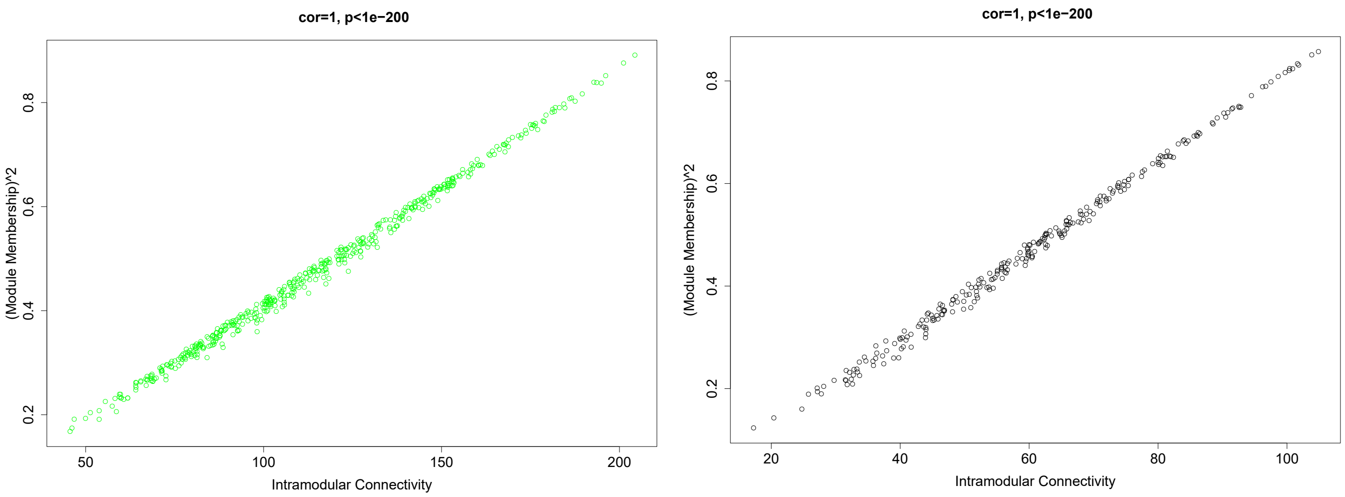

B

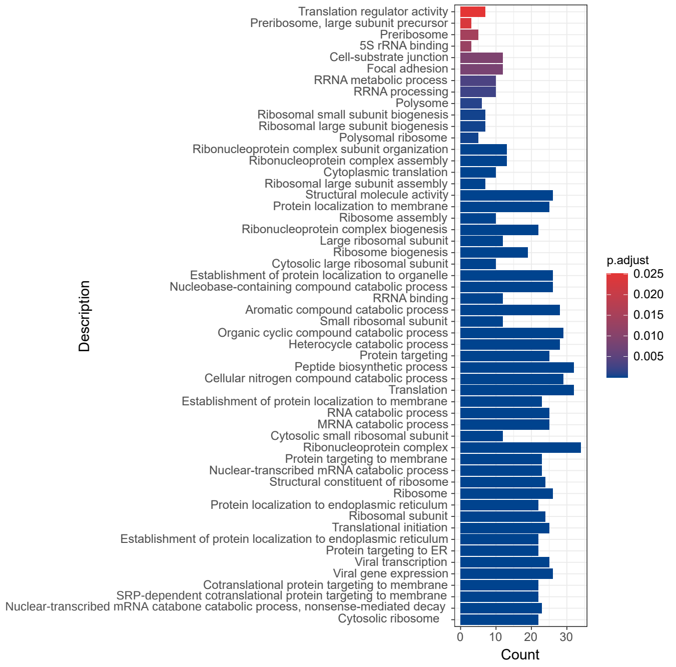

C

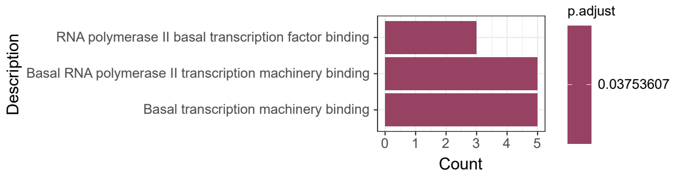

Figure S2. (A) Intramodular connectivity measures connectivity or co-expression of given genes in the MEgreen and the MEblack modules. **(B)** GO enrichment analysis of the genes negatively correlation with CSA of H, D and HD. **(C)** GO enrichment analysis of the genes positively correlation with CSA of H, D and HD.
