# Supplementary material for: Dynamic Changes in the Global Transcriptome of Postnatal Skeletal Muscle in Different Sheep
Source: Genes (Basel). 2023 Jun 20;14(6):1298. doi: 10.3390/genes14061298 (PMC10297920; doi:10.3390/genes14061298)
Supplement: Supplementary file 1 [file genes-14-01298-s001.zip › FigureS3.pdf]

Figure. S3

A

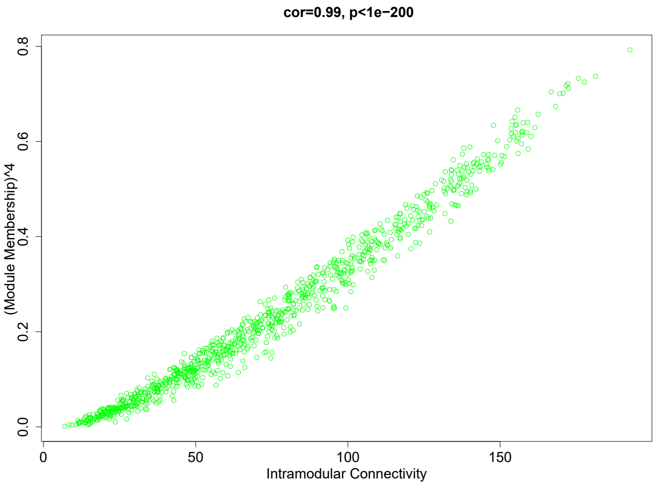

B

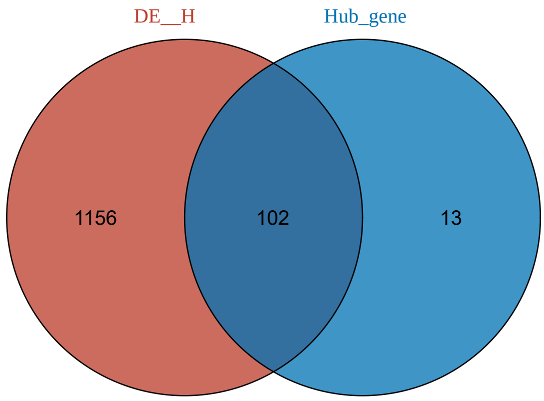

C

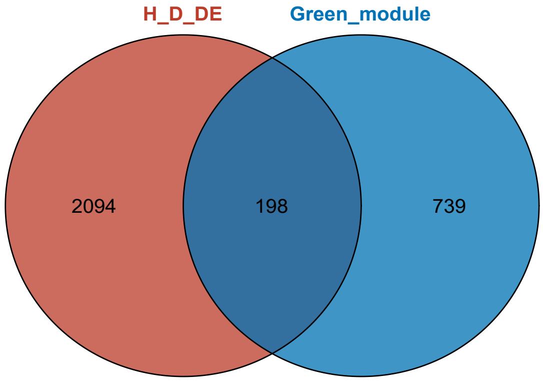

D

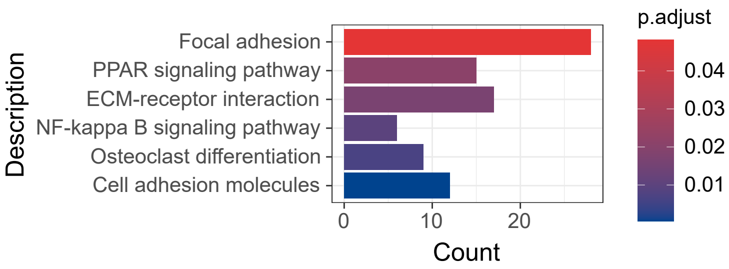

Figure S3.(A) Intramodular connectivity measures connectivity or co-expression of given genes in the MEgreen modules. **(B)** The DEGs of H and MEgreenmodule hub gene in Figure3B intersection gene.**(C)** The DEGs between H and D from 3M to 12M and the MEgreen module in Figure3B intersection gene. **(D)** KEGG pathways for Figure3E genes.
