# Supplementary figures and images for: Dynamic Changes in the Global Transcriptome of Postnatal Skeletal Muscle in Different Sheep
Source: Genes (Basel). 2023 Jun 20;14(6):1298. doi: 10.3390/genes14061298 (PMC10297920; doi:10.3390/genes14061298)

Figure. S1

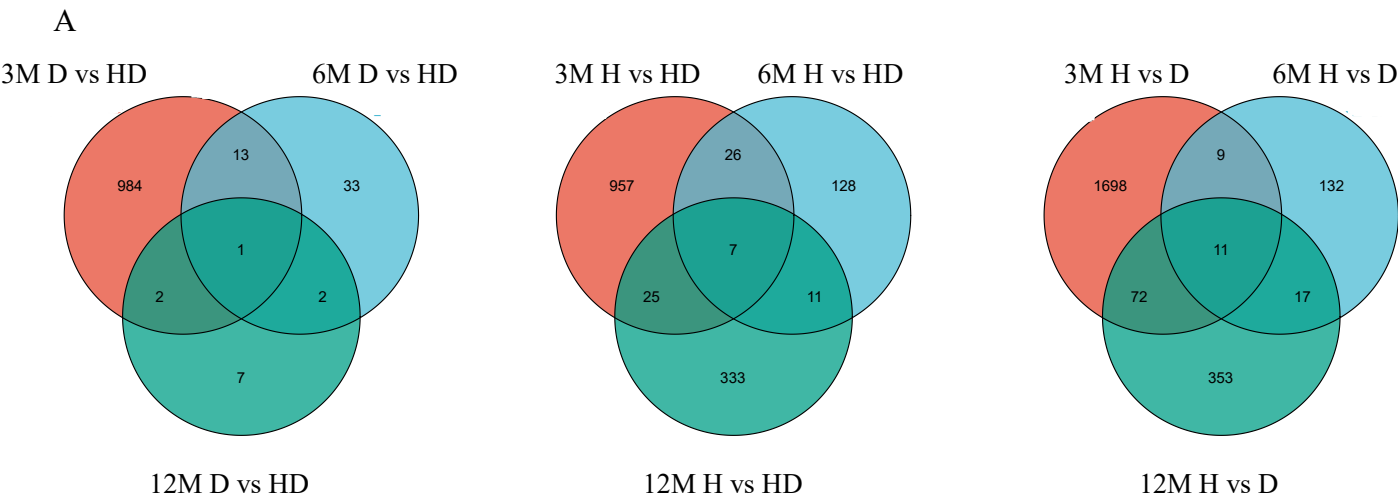

Figure S1. Venn diagram showed stage-specific DEGs for any pair of three breeds.

Supplement: Supplementary file 1 [file genes-14-01298-s001.zip › FigureS1.pdf]
